# Aging Alters the Formation and Functionality of Signaling Microdomains Between L-type Calcium Channels and β2-Adrenergic Receptors in Cardiac Pacemaker Cells

**DOI:** 10.3389/fphys.2022.805909

**Published:** 2022-04-20

**Authors:** Sabrina Choi, Oscar Vivas, Matthias Baudot, Claudia M. Moreno

**Affiliations:** Department of Physiology and Biophysics, University of Washington, Seattle, WA, United States

**Keywords:** cardiac pacemaker, aging, L-type calcium channel, beta-adrenergic receptor, signaling microdomain, AKAP150, caveolin-3 (Cav-3), scaffolding proteins

## Abstract

Heart rate is accelerated to match physiological demands through the action of noradrenaline on the cardiac pacemaker. Noradrenaline is released from sympathetic terminals and activates β1-and β2-adrenergic receptors (ΑRs) located at the plasma membrane of pacemaker cells. L-type calcium channels are one of the main downstream targets potentiated by the activation of β-ARs. For this signaling to occur, L-type calcium channels need to be located in close proximity to β-ARs inside caveolae. Although it is known that aging causes a slowdown of the pacemaker rate and a reduction in the response of pacemaker cells to noradrenaline, there is a lack of in-depth mechanistic insights into these age-associated changes. Here, we show that aging affects the formation and function of adrenergic signaling microdomains inside caveolae. By evaluating the β1 and β2 components of the adrenergic regulation of the L-type calcium current, we show that aging does not alter the regulation mediated by β1-ARs but drastically impairs that mediated by β2-ARs. We studied the integrity of the signaling microdomains formed between L-type calcium channels and β-ARs by combining high-resolution microscopy and proximity ligation assays. We show that consistent with the electrophysiological data, aging decreases the physical association between β2-ARs and L-type calcium channels. Interestingly, this reduction is associated with a decrease in the association of L-type calcium channels with the scaffolding protein AKAP150. Old pacemaker cells also have a reduction in caveolae density and in the association of L-type calcium channels with caveolin-3. Together the age-dependent alterations in caveolar formation and the nano-organization of β2-ARs and L-type calcium channels result in a reduced sensitivity of the channels to β2 adrenergic modulation. Our results highlight the importance of these signaling microdomains in maintaining the chronotropic modulation of the heart and also pinpoint the direct impact that aging has on their function.

## Introduction

**FIGURE 1:**
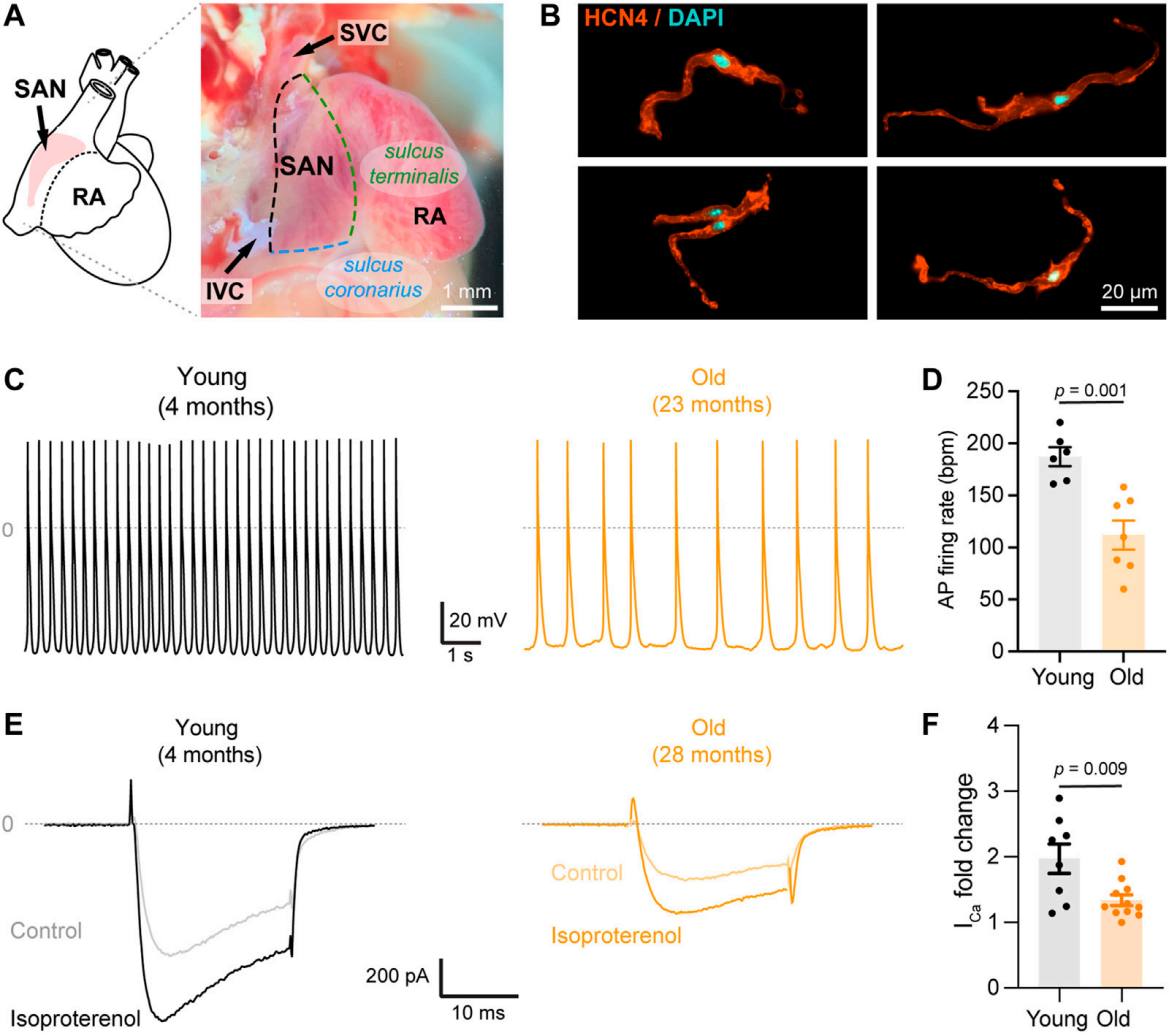
Aging slows down action potential firing and decreases isoproterenol-mediated up regulation of calcium currents. **(A)** (*left*) Schematic representation of the position of the SAN pacemaker in the heart; (right) Image of the intercaval region of the mouse heart depicting the anatomical landmarks used to isolate the pacemaker explant. **(B)** Representative confocal images of isolated pacemaker cells immunostained against HCN4 (orange) and counterstained with DAPI (cyan). **(C)** Representative recordings of spontaneous firing of action potential from a young (gray) and old (orange) isolated pacemaker cell. **(D)** Comparison of the action potential firing rate in beats per minute (bpm) in young (n = 6, N = 3 animals) and old (n = 7, N = 4 animals) pacemaker cells. **(E)** Representative calcium currents from young (*left*, gray) and old (*right,* orange) cells, in the absence (light) or presence (dark) of 100 nM isoproterenol. **(F)** Fold-change of the calcium current peak after isoproterenol application in young (black, n = 10 cells, N = 3 animals) and old (orange, n = 12 cells, and N = 5 animals) pacemaker cells. Statistical comparison used an unpaired t-test with significance at *p* < 0.05.

Animals regulate their heart rate to match physiological demands and to respond to external challenges such as danger. The homeostatic regulation of heart rate is achieved through fine neural control of the cardiac pacemaker. Anatomically known as the sinoatrial node (SAN), the cardiac pacemaker is a specialized tissue located at the intercaval region (Figure 1A) and has the ability to spontaneously generate the electricity that initiates each heartbeat. Despite occupying less than 2% of the total volume of the heart, the SAN is the most innervated region in this organ (Csepe et al., 2016). Autonomic terminals innervate the pacemaker and regulate its firing rate through the release of neurotransmitters. Pacemaker rate is accelerated by the action of noradrenaline released from sympathetic terminals (Brown et al., 1979; DiFrancesco, 1986; Boyett et al., 2000; Mangoni and Nargeot, 2008). Noradrenaline activates beta-adrenergic receptors (β-ARs) expressed in the surface of pacemaker cells and triggers a signaling pathway that elevates cAMP levels and results in firing rate acceleration.

Mammals experience a natural slowdown of the intrinsic pacemaker rate throughout their lifespan (Yin et al., 1979; Di Gennaro et al., 1987; Marcus et al., 1990; Alings and Bouman, 1993; Ostchega et al., 2011). Pacemaker rate slowdowns linearly with age at a rate of ∼0.8 bpm/year in humans (Tanaka et al., 2001) and ∼4 bpm/month in mice (Larson et al., 2013). This slowdown is the main cause for the development of pathological SAN dysfunction and for the requirement of artificial pacemaker devices in humans. The slowdown of the pacemaker has been proposed to be caused by a combination of mechanisms including the reduction in the activation of HCN channels (Larson et al., 2013; Sharpe et al., 2017), the loss of pacemaker cells (Evans and Shaw, 1977; Thery et al., 1977; Shiraishi et al., 1992), and an increase in tissue fibrosis (Evans and Shaw, 1977; Thery et al., 1977; Shiraishi et al., 1992; Hao et al., 2011).

Interestingly, the age-associated slowdown of the pacemaker is accompanied by a reduction in its sensitivity to adrenergic stimulation (Yaniv et al., 2016; Alghamdi et al., 2020). It is estimated that about 17% of the age-associated decrease in maximum heart rate is explained by a loss of the absolute response of pacemaker cells to adrenergic modulation (Christou and Seals, 2008; Peters et al., 2020). However, our understanding of how aging affects adrenergic signaling in pacemaker cells is still incomplete. So far, we know that aging causes a reduction in the expression of some electrogenic proteins including HCN4, Ca_V_1.2, Na_V_1.5, and several K^+^ channels (Jones et al., 2007; Tellez et al., 2011; Alghamdi et al., 2020). However, dialysis of a high concentration of exogenous cAMP into pacemaker cells completely restores action potential firing rate in old cells to the same levels observed in young cells (Sharpe et al., 2017), suggesting that in old cells, the machinery responsible for the adrenergic pathway retains its full potential to be activated. If these proteins are fully functional, then what prevents them from operating at full capacity? We explore the idea that aging disrupts the spatial organization of some components of the adrenergic signaling pathway.

The automaticity of the pacemaker relies on a spontaneous phase known as diastolic depolarization which brings the membrane potential to the required threshold to trigger the action potential (AP). This diastolic depolarization is sustained by the activation of HCN4 channels (Baruscotti et al., 2010), voltage-gated calcium channels (Furukawa et al., 1999; Wang et al., 2000), ryanodine receptors (RyR) (Vinogradova and Lakatta, 2009; MacDonald et al., 2020), and the calcium sodium exchanger (NCX) (Ebert and Taylor, 2006; Mangoni and Nargeot, 2008; Gordan et al., 2015). L-type calcium channels play an essential role in the normal function of the pacemaker (Christel et al., 2012). Ca_V_1.3 channels contribute to the diastolic depolarization phase and the potentiation of calcium release from RyR (Torrente et al., 2016). The important role of Ca_V_1.3 channels in the control of pacemaker rate is highlighted by the fact that knockout animals exhibit bradycardia (Mangoni et al., 2003; Baudot et al., 2020), and mutations of this channel are associated with sick sinus syndrome in humans (Milanesi et al., 2015). Ca_v_1.2 channels are essential for sustaining the AP since pacemaker cells express very low amounts of voltage-gated sodium channels compared to atrial and ventricular cardiomyocytes (Mangoni and Nargeot, 2008; Christel et al., 2012).

Upregulation of L-type calcium channels is one of the crucial mechanisms by which noradrenaline accelerates the pacemaker rate (Zaza et al., 1996). The cAMP elevation caused by the activation of β-ARs increases L-type calcium channel activity through two known mechanisms: the first is direct PKA-mediated phosphorylation of the α subunit of the channel (Fu et al., 2014), and the second is the removal of the tonic inhibition of the channel mediated by the small GTPase Rad (Liu et al., 2020; Levitan et al., 2021). Pacemaker cells express β1-and β2-AR as other cardiac cells; however, the pacemaker is the region with the highest β2 expression in the heart (Brodde et al., 1982; Rodefeld et al., 1996). The expression ratio in the pacemaker is 51.6% *±* 3.2 for β1-AR and 48.4% *±* 3.2 for β2-AR (Saito et al., 1989) compared to the 70:30 observed in atrial and the 80:20 in ventricular cells (Brodde et al., 1982; Brodde et al., 2001). Evidence in ventricular cardiomyocytes shows that L-type calcium channels associate preferentially with β2-AR inside T-tubules (Nikolaev et al., 2006) and caveolae (Shibata et al., 2006; Harvey and Hell, 2013). Although pacemaker cells lack T-tubules, it has been proposed that in these cells, caveolae serve as the compartments for the signaling of β2-ARs with ion channels. Although evidence exists for the association of caveolin-3 with channels (Barbuti et al., 2007), no evidence for the association with LTCCs has been provided in pacemaker cells. Furthermore, whether the age-associated reduction in the sensitivity of the pacemaker to adrenergic stimulation involves changes in these signaling microdomains remains unknown.

Here, we tested the hypothesis that aging differentially affects the association of L-type calcium channels with β1 and β2-ARs and the upregulation mediated by each subtype. Our results reveal that aging specifically impairs the L-type calcium channel upregulation mediated by β2-ARs. This effect is accompanied by a reduction in caveolar density and in the association of L-type calcium channels with caveolin-3 and AKAP150. Our results highlight the importance of these signaling microdomains in maintaining the chronotropic modulation of the heart and unveil a new mechanism behind the age-associated loss of sensitivity to adrenergic modulation in pacemaker cells.

## Methods

### Isolation of Pacemaker Cells

Pacemaker cells were freshly isolated from young (4–6 months, equivalent to ∼20–30 years in humans) and old (20–24 months, equivalent to ∼60–69 years in humans) C57BL/6 male mice. Animals were anesthetized with an intraperitoneal overdose of Euthasol (Virbac, 400 mg/ml), and their hearts were harvested according to a protocol approved by the UW Institutional Animal Care and Use Committee (IACUC). The harvested heart was dissected under the microscope in warm Tyrode’s solution containing: 148 mM NaCl, 5.4 mM KCl, 5 mM HEPES, 5.5 mM glucose, 1 mM MgCl_2_, and 1.8 mM CaCl_2_, adjusted to pH 7.4 with NaOH. The sinoatrial node (SAN) region was identified as the region located within the superior vena cava, the *sulcus terminalis,* the *coronary sulcus,* and the inferior vena cava (Figure 1A). The SAN artery was also used as an anatomical landmark to identify the pacemaker. Pacemaker cells were isolated from the SAN tissue as described in Fenske et al. (2016). Briefly, the excised SAN tissue was immersed into a pre-heated 2 ml tube at 37°C containing 675 μl of Tyrode Low-Ca^2+^ pH 6.9 solution for 5 min. Then BSA was added to the tube to obtain a final concentration (Cf) of 1 mg/ml, followed by the addition of elastase (Cf = 18.87 U/ml, Millipore 3,24,682), protease (Cf = 1.79 U/ml, Sigma P5147), and collagenase B (Cf = 0.54 U/ml, Roche 11088807001). Digestion was carried out for 30 min at 37°C in a water bath along with a mechanical dissociation with a short fire-polished glass pipette every 10 min. To stop the digestion, the SAN was washed by centrifugation at 200 rfc for 2 min at 4°C. The supernatant was discarded and replaced twice with 1 ml of Tyrode’s Low-Ca^2+^ solution containing: 140 mM NaCl, 5.4 mM KCl, 5 mM HEPES, 5.5 mM glucose, 1.2 mM KH_2_PO_4_, 50 mM taurine, 0.5 mM MgCl_2_, and 0.2 mM CaCl_2_, adjusted to pH 6.9 with NaOH. This process was repeated 2 more times with 1 ml of calcium-free KB solution containing: 80 mM L-glutamic acid, 25 mM KCl, 10 mM HEPES, 10 mM glucose, 10 mM KH_2_PO_4_, 20 mM taurine, 0.5 mM EGTA, and 3 mM MgCl_2_, adjusted to pH 7.4 with KOH. The tissue was left resting in KB solution at 4°C for a minimum of 40 min before proceeding to mechanical dissociation. Single cells were dissociated by mechanical dissociation as aforementioned. The concentration and incubation times for the enzymes were slightly modified to obtain cells from old animals. In this case, the SAN explant was incubated for 20 min in Tyrode’s Low-Ca^2+^ containing twice the concentration of collagenase B, followed by the same 30 min digestion with the three enzymes at the concentration used for young tissues. Mechanical dissociation for the old tissue was performed every 5–7 min for the duration of the incubation. Cells were plated on poly-L Lysine (PLL)-coated coverslips for immunocytochemistry or PLA assays. For electrophysiology experiments, to recover the automaticity of young and old pacemaker cells, Ca^2+^ was reintroduced into the KB cell’s storage solution by the gradual addition of small amounts of Tyrode’ solution (10, 50, and 100 μl at 5 min intervals).

### Electrophysiology Recordings

Action potentials were recorded using perforated patch-clamp under gap-free acquisition. Borosilicate patch pipettes with resistances of 3–5 MΩ were filled with an intracellular solution containing 130 mM L-Aspartic acid K, 10 mM NaCl, 0.04 mM CaCl_2_, 10 mM HEPES, 2 mM Mg-ATP, 0.1 mM Na-GTP, 6.6 mM phosphocreatine, and adjusted to pH 7.2 with KOH. Freshly prepared 25 μM β-Escin were added to the internal solution the day of the experiment. Cells were perfused at room temperature with Tyrode’s solution containing 140 mM NaCl, 5.4 mM KCl, 1 mM MgCl_2_, 1.8 mM CaCl_2_, 5 mM HEPES, and 5.5 mM Glucose, adjusted to pH 7.4 with NaOH. Seal resistances were in the range of 2–5 GΩ, and no holding or transient current was applied. Data acquisition was performed using an Axoclamp 200B patch-clamp amplifier connected to a Digidata 1,500 interface (Molecular Devices).

Calcium currents were recorded using the whole-cell configuration of the patch-clamp technique in voltage-clamp mode. Isolated pacemaker cells were perfused with Tyrode’s solution at room temperature before starting recordings. Borosilicate patch pipettes with resistances of 3–4 MΩ were filled with an internal solution containing: 50 mM CsCl, 10 mM HEPES, 70 mM L-Aspartic acid, 30 mM TEA-Cl, 5 mM EGTA, 5 mM Mg-ATP (added right before use), 1 mM MgCl2, and 0.7 mM CaCl2, adjusted to pH 7.2 with CsOH. Once the gigaseal was obtained, the bath solution was exchanged from Tyrode’s to a solution without sodium, containing: 5 mM CsCl, 10 mM HEPES, 10 mM glucose, 110 mM N-methyl-D-glucamine, 30 mM TEA-Cl, 4 mM 4-Aminopyridine, 1 mM MgCl2, and 2 mM CaCl2, adjusted to pH 7.4 with HCl. Cells were then stimulated using a 20 ms square voltage pulse from a resting membrane potential of −70 mV to −20 mV. Currents were sampled at a frequency of 10 kHz and low-pass filtered at 2 kHz using an Axopatch 200B amplifier. To activate β2-ARs and β1-ARs in a sequential manner, we used the specific β2 agonist, formoterol, which is 330-fold more selective for β2 than for β1. An external solution with 100 nM of formoterol was perfused first (β2-AR activation), followed by a solution with 100 nM formoterol +100 nM isoproterenol (β2-AR + *β*1-AR activation). Upregulation induced by β1-AR was calculated by subtracting these two components. Lastly, a solution containing 10 μM of nifedipine was perfused at the end of the experiments to block the L-type calcium channels. Currents were analyzed using pCLAMP 11 (Molecular Devices).

### Protein Extraction and Western Blot

SAN explants dissected using the landmarks shown in Figure 1A were homogenized in RIPA lysis buffer (Thermo Scientific, Cat # 89,900) and supplemented with Complete Mini protease inhibitor cocktail (Roche, Cat # 11836170001). After centrifugation (16,000g, 4°C, and 20 min), the concentration of protein lysates in the supernatant was determined using Pierce BCA Protein Assay Kit (Thermo Scientific, Cat # 23,225). 40 μg of total protein were loaded per line on 4–12% polyacrylamide Bis-Tris gels, run under reducing conditions for 1 h 15 min at 155 V, and transferred onto nitrocellulose membranes (Life Technologies, Cat # LC2000) using a Mini-Bolt system (A25977; Thermo Fisher Scientific). After 1 h of incubation at room temperature in TBS buffer supplemented with 0.05% Tween-20 (TBS–T) and 7% non-fat dry milk, membranes were exposed overnight at 4°C to rabbit monoclonal β2-AR antibody (Abcam Cat# ab182136, RRID: AB_2747383), goat polyclonal β1-AR antibody (Abcam Cat# ab77189, RRID: AB_1523202), rabbit polyclonal β1-AR antibody (Abcam Cat# ab3442), or mouse monoclonal GAPDH antibody (Abcam Cat# ab8245, RRID: AB_2107448). Membranes were washed in TBS-T and incubated for 1 h at room temperature with secondary antibodies. The secondary antibody against rabbit β1-AR and β2-AR were HRP-conjugated. Secondary antibodies against goat β1-AR and GAPDH were fluorescent. Fluorescent blotted bands were detected using fluorescent secondary antibodies donkey anti-goat 680RD (LI-COR Biosciences Cat# 925-68,074, RRID: AB_2650427) and donkey anti-mouse 800CW (LI-COR Biosciences Cat# 926-32212, RRID: AB_621847). Signals were detected using either an iBright imaging system (Thermo Fisher Scientific) for chemiluminescent or a Sapphire Gel Imager (Azure Biosystems) for fluorescent blots. ImageJ was used to calculate the fluorescence density of each band. β2-AR bands were normalized to total protein, while β1-AR bands were normalized to GAPDH (N = 2) or total protein (N = 5). Total protein was detected using the No-stain® reagent (A44449 ThermoFisher). Protein abundance was reported relative to the abundance in young pacemaker explants.

### Proximity Ligation Assay

12 mm diameter round 1.5 glass coverslips were coated with PLL hydrobromide (Sigma P1524, MW ≥ 300,000) for 30 min at 37°C; then, thoroughly washed with water, and left to dry overnight. Isolated pacemaker cells were plated on these coverslips and left to attach for 30 min at 37°C in a humidity chamber. Cells were fixed with 4% paraformaldehyde in PBS for 10 min at room temperature and washed with PBS. All the washing steps in this protocol, unless stated otherwise, consisted of 3 rinses with PBS followed by 3 × 5 min washes with PBS on a rocker. To quench any excess PFA, 50 mM Glycine in PBS was added for 15 min at room temperature and washed with PBS. Cells were then blocked with Duolink Blocking Solution (Sigma, DUO82007) for 30 min at 37°C in a humidity chamber. Primary antibodies were diluted in Duolink Antibody Diluent Solution (Sigma, DUO82008) to a concentration of 10 μg/ml (100 μl/coverslip) and incubated overnight at 4°C under gentle orbital agitation. We used the following antibodies: Ca_V_1.2 and Ca_V_1.3 (rabbit anti CNC1 and CND1, respectively; provided by Drs. William Catterall and Ruth Westenbroek, UW), mouse anti-β2-AR (Santa Cruz Biotechnology, sc-271322), goat anti-β1-ARs (Abcam, ab77189), rabbit anti-β1-ARs (Abcam, ab3442), mouse anti-caveolin-3 (BD Transduction Labs, 610420), and mouse anti-AKAP150 (BD Transduction Labs, A31320G). The following day, coverslips were washed with PBS and transferred to a parafilm-lined humid chamber for easier application of solutions. Coverslips were rinsed twice with 100 μl of Duolink Buffer A (Sigma, DUO82049) for 5 min under gentle orbital agitation. Rabbit-plus and mouse-minus or goat-minus Duolink *In Situ* PLA secondary probes (Sigma DUO92002; 92004; 92006) were added according to manufacturer’s specifications and incubated for 1 h at 37°C in a humidity chamber. This was followed by the addition of 20 μl per coverslip of ligation solution consisting of 4 μl 5X ligation stock, 15.5 μl pure water, and 0.5 μl ligase (Duolink *In Situ* PLA Detection Reagents Orange, Sigma DUO92007) for 30 min at 37°C in a humidity chamber. 20 μl of amplification solution consisting of 4 μl 5X amplification stock, 15.75 μl pure water, and 0.25 μl polymerase (Duolink *In Situ* PLA Detection Reagents Orange, Sigma DUO92007), was then added to each coverslip and incubated for 100 min at 37°C in a humidity chamber, followed by two 10 min Duolink Buffer B (Sigma, DUO82049) washes and 1 min Duolink Buffer B (1%) wash under gentle orbital agitation. Coverslips were left to dry covered from light for a minimum of 30 min before mounting on microscope slides with Prolong Diamond Antifade Mountant (no DAPI, Invitrogen, P36961). Images were taken as described below but with these additional experimental details. Z-stacks with a step size of 0.5 μm were acquired to generate high-resolution images. Images were processed with ImageJ (NIH). Processing consisted of filtering with a Median 3D filter with a sigma of 1 for each axis, converting the stack into a maximum intensity projection, thresholding, and binarization. Finally, images were analyzed to calculate cell area and the number of particles from these Z-projections. Data is reported as particle density for each cell.

### Immunocytochemistry and Expansion Microscopy

For HCN4 and caveolin-3 immunostaining, pacemaker cells plated on 12 mm PLL-coated coverslips were fixed in 4% PFA in PBS for 15 min. All the washing steps in this protocol, unless stated otherwise, consisted of 3 rinses with PBS followed by 3 × 5 min washes with PBS on a rocker. After washing with PBS, cells were incubated with 1 mg/ml NaHB_4_ at room temperature for 5 min and washed again with PBS. Cells were blocked by incubating with 3% BSA and 0.25% v/v Triton X-100 in PBS (blocking solution) for 30 min. The cells were then incubated for 1 h at room temperature with mouse anti-caveolin-3 antibody (BD Transduction Labs, 6,10,420) or overnight at 4°C with rabbit anti-HCN4 (Sigma, AB5808) at 10 μg/ml in blocking solution. Cells were then washed with PBS, incubated for 1 h with Alexa Fluor 488-conjugated donkey anti-mouse or donkey anti-rabbit (2 μg/ml; Invitrogen, A32787/A32790) secondary antibodies, and washed again with PBS. For immunocytochemistry, coverslips were mounted using Prolong Diamond Antifade Mountant (with DAPI, Thermo Scientific, P36962).

For expansion microscopy, we closely followed the protocol published by Chozinski et al. (2016). Instead of mounting, the immunostained coverslips were incubated with 25 mM MA-NHS in PBS for 1 h at room temperature, followed by 3 washes with PBS. The coverslips were incubated in a monomer solution of 2 M NaCl, 2.5% (w/w) acrylamide, 0.15% (w/w) N, N′-methylenebisacrylamide, and 8.625% (w/w) sodium acrylate, for ∼1 min at room temperature prior to gelation. Concentrated stocks of tetramethylethylenediamine (TEMED) and ammonium persulfate (APS) at 10% (w/w) in water were freshly prepared. TEMED and APS were quickly added to the monomer solution to achieve a final concentration of 0.2% (w/w). 70 µl of the gelation solution were placed on a Teflon flat surface and the coverslip was then placed on top of this solution with cells face down. Gelation was allowed to proceed at room temperature for 30 min. The coverslip and gel were removed with tweezers and placed in digestion buffer (1 × TAE buffer, 0.5% Triton X-100, 0.8 M guanidine HCl) containing 8 units/ml of freshly added Proteinase K (Thermo Scientific, EO0491). Gels were digested at 37°C overnight. The next day, the gels were placed in ∼50 ml DI water to expand. Water was replaced every 30 min x 4 or until expansion was complete. Cells were expanded 2.5 times and the resolution was 50 nm.

### High-Resolution Imaging

Cells were imaged using an inverted AiryScan microscope (Zeiss LSM 880) run by Zen black v2.3 and equipped with a plan apochromat 63X oil immersion lens with a 1.4 NA. Fluorescent dyes were excited with a 405 nm diode, 458–514 nm argon, 561 nm, or 633 nm laser. Emission light was detected using an Airyscan 32 GaAsP detector and appropriate emission filter sets. The point spread functions were calculated using ZEN black software and 0.1 μm fluorescent microspheres. After deconvolution, the point spread functions were: 124 nm in X-Y, and 216 nm in Z (488 nm excitation); 168 nm in X-Y and 212 nm in Z (594 nm excitation). The temperature inside the microscope housing was 27–30°C. Images were analyzed using a custom-made macro written in ImageJ (NIH). Processing consisted of thresholding and binarization of images to isolate labeled structures. Particles were analyzed to calculate the area and number of particles from these images.

## Results

### Aging Slows Down Action Potential Firing and Decreases Adrenergic Up Regulation of Calcium Currents in Pacemaker Cells

It is known that aging reduces the adrenergic-mediated acceleration of heart rate and the sensitivity of pacemaker cells to the non-specific agonist, isoproterenol (Christou and Seals, 2008; Larson et al., 2013; Yaniv et al., 2016). Despite calcium channels being one of the main targets of β-adrenergic modulation, there are only a few studies on the effects of aging on the isoproterenol-induced upregulation of calcium currents (Dun et al., 2003; Larson et al., 2013). We tested the effect of isoproterenol in the upregulation of calcium-currents in pacemaker cells isolated from the SAN. Isolated cells presented the morphology reported for pacemaker cells and were positive for the pacemaker marker HCN4 (Figure 1B), confirming the identity of the cells. In addition, current-clamp recordings demonstrated the automaticity of the isolated pacemaker cells (Figure 1C). As previously reported, aging slowed the action potential firing rate (Figure 1D). While young cells fired action potentials (AP) at a rate of 187 ± 9 beats per minute (bpm, n = 6 cells), old cells fired AP at a rate of 112 ± 14 bpm (n = 7 cells). Moreover, old pacemaker cells exhibited different parameters of the action potentials. For example, the cycle length was 624 ± 97 ms in old cells compared to 324 ± 15 ms in the young, action potentials last for 260 ± 23 ms in old cells compared to 183 ± 24 ms in the young, and the diastolic depolarization lasted for 314 ± 73 ms in old cells compared to 127 ± 9 ms in the young. All quantified parameters, n number, and *p*-values are shown in Supplementary Table S1. We recorded time courses of calcium currents in isolated pacemaker cells from young and old animals before and during the application of 100 nM isoproterenol. The application of isoproterenol was efficient in upregulating the calcium current in young and old cells (Figure 1E). Isoproterenol upregulated the calcium current in the young group by 2-fold (n = 8), and by 1.3-fold in the old (n = 11, Figure 1F), suggesting that aging significantly reduces the isoproterenol-mediated upregulation of calcium currents in pacemaker cells (*p* = 0.009).

### Aging Reduces β2-but Not β1-Mediated Upregulation of the L-type Calcium Current

**FIGURE 2:**
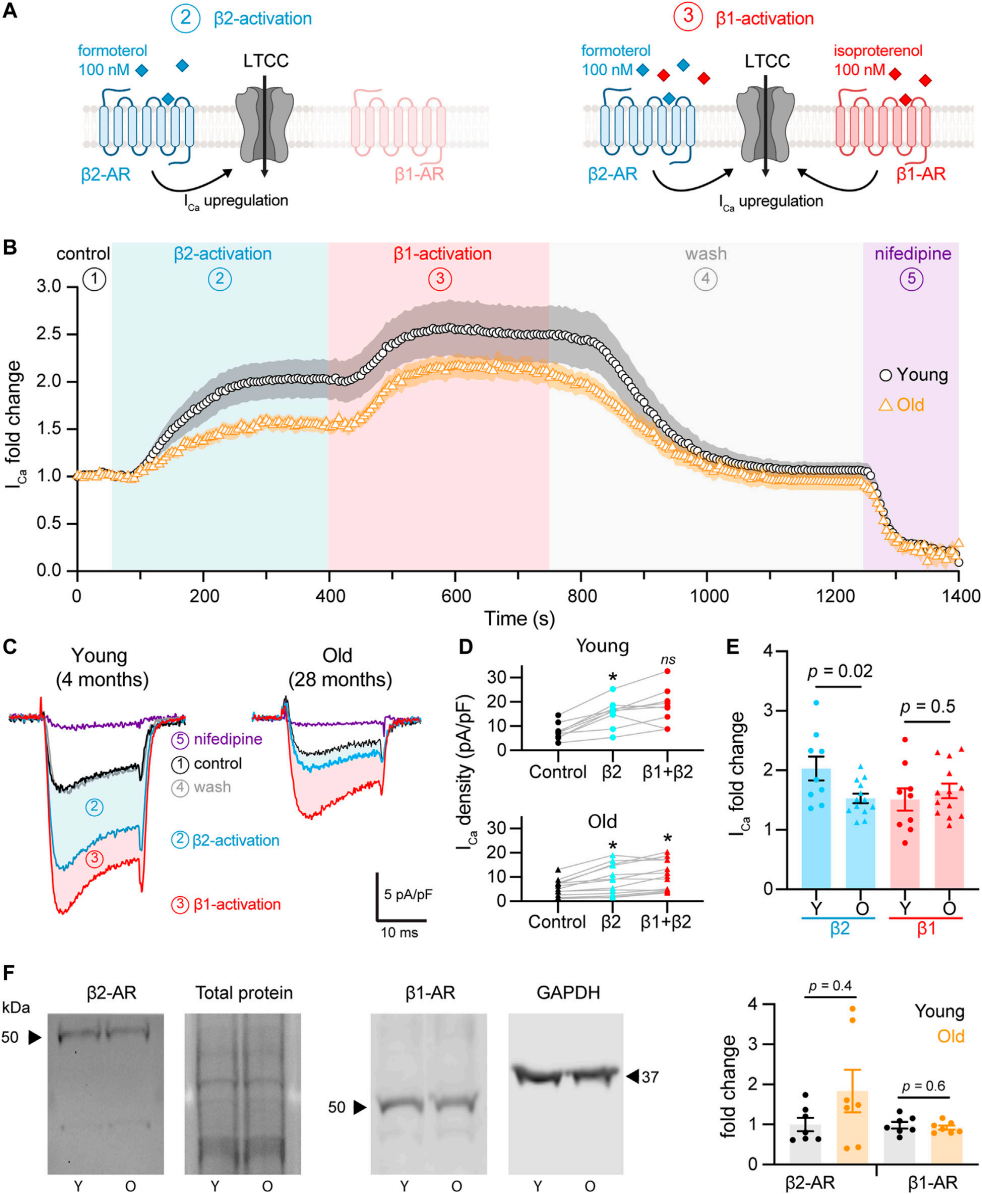
Aging reduces β2-but not β1-mediated regulation of L-type calcium currents in pacemaker cells. **(A)** Diagrammatic representation summarizes the protocol used to isolate β2 from β1-AR upregulation of calcium currents, which consisted in the stimulation using 100 nM formoterol followed by the simultaneous stimulation with 100 nM formoterol +100 nM isoproterenol. Diagram created with BioRender.com. **(B)** Time course of calcium current fold change in control conditions (1) β2-specific activation (2) β1-activation (3), washout (4), and lastly with nifedipine to block LTCCs (5) in young (black circles, n = 10 cells, N = 3 animals) and old (orange triangles, n = 14 cells, N = 4 animals) pacemaker cells. **(C)** Representative calcium currents from the time course in B. **(D)** Calcium current density regulation after the activation of β2-and co-activation of β2 and β1-ARs relative to control and paired for individual cells isolated from young (top) and old (bottom) mice. Statistical comparison relative to the control condition used two independent paired t-tests with significance at *p* < 0.05. **(E)** Comparison of the fold-change of the calcium current peak after the activation of β2 and β1-ARs from the same cells presented in D. Statistical comparison used two independent unpaired t-tests with significance at *p* < 0.05. **(F)** Western blots testing for changes in the expression of β2 and β1-ARs from SAN explants. β2-AR expression was normalized to total protein (N = 7 independent pacemaker isolations per age, *p* = 0.4), while β1-AR expression was normalized to GAPDH in 2 of the 7 experiments and to total protein in 5 of the 7 experiments (N = 7 independent pacemaker isolations per age, *p* = 0.6).

As we mentioned before, pacemaker cells express in similar proportions β1 and β2-AR. We already showed that aging reduces the global adrenergic upregulation of calcium channels. However, whether the β1 and β2 components are differentially affected by aging is not known. We tested the hypothesis that aging has a differential effect on the upregulation of calcium currents triggered by the activation of either β1 or β2-AR. For this, we designed an experimental approach to study upregulation of the calcium current in pacemaker cells under a 5 min application of the β2-specific agonist formoterol (100 nM) followed by a 5 min simultaneous application of formoterol (100 nM) and isoproterenol (100 nM) to activate the remaining β1 component (Figure 2A). We stimulated the cells with a voltage step from −70 to −20 mV every 5 s and measured the change in peak of current density over time. As shown in the average temporal course in Figure 2B and in the representative examples in Figure 2C, calcium currents in both young and old cells were upregulated under the activation of the β2-ARs (phase 2) and further upregulated upon the activation of the β1-ARs (phase 3). The upregulation of the calcium current was completely reversed by washing off the agonists and the current amplitude was restored to basal levels (phase 4 in Figures 2B,C). The calcium current was mainly carried by L-type calcium channels as confirmed by the application of 10 μM nifedipine, which abolished about 80% of the current in both young and old cells (phase 5 in Figures 2B,C).

We quantified the change in the calcium current upon β2 and β1-AR activation. Figure 2D shows the change in the raw current density for each cell. β2-AR activation increased calcium current density in young and old cells, going from 7.8 ± 1.1 pA/pF to 15.4 ± 1.9 pA/pF in the young group (*p* = 0.0007) and from 5.4 ± 1.0 pA/pF to 8.7 ± 1.7 pA/pF in the old (*p* = 0.006, Figure 2D). Subsequent activation of β1-AR in the same cells led to a further upregulation reaching a maximal current density of 18.2 ± 2.5 pA/pF in the young (*p* = 0.2) and 10.7 ± 1.7 pA/pF in the old group (*p* = 0.02). To compare the regulation from each receptor, we calculated the fold-change relative to the peak of the current in control conditions. Interestingly, the β2-mediated upregulation was significantly reduced in old cells. The activation of β2-ARs increased the calcium current 2.0 ± 0.2 times relative to control levels in young cells (n = 9), whereas it only increased it 1.5 ± 0.1 times in old cells (n = 13) (Figure 2E). In contrast, the activation of β1-ARs increased the calcium current 1.5 ± 0.2 times in young (n = 9) and 1.6 ± 0.1 times in old (n = 13) (Figure 2E). These results suggest that aging causes a significant reduction in the sensitivity of the calcium current to the activation of β2-ARs, but not of β1-ARs.

Next, we assessed if old cells had a reduction in the expression of β2-ARs that could account for the observed reduction in the β2-mediated regulation. Using protein extracts from young and old pacemaker explants, we did not find any difference in β2-ARs expression (Figures 2F,G). The average fold change for β2-ARs in old compared to young pacemakers was 1.8 ± 0.5 (N = 7 animals per age) independent pacemaker isolations, *p* = 0.4). Expression of β1-ARs was also unchanged. The average fold change for β1-ARs in old compared to young pacemakers was 0.9 ± 0.05 (N = 7 animals per age). An important consideration regarding this experiment is that even though only the SAN explant was collected, the tissue contains different cell types (i.e., vascular cells, neurons, fibroblasts). As a consequence, the analyzed abundance of β-ARs reflected the expression in all cell types. These results suggest that aging reduces β2-mediated upregulation by a mechanism independent of receptor level changes.

### The Physical Association Between L-type Calcium Channels and β2-AR Is Reduced in Old Pacemaker Cells

**FIGURE 3:**
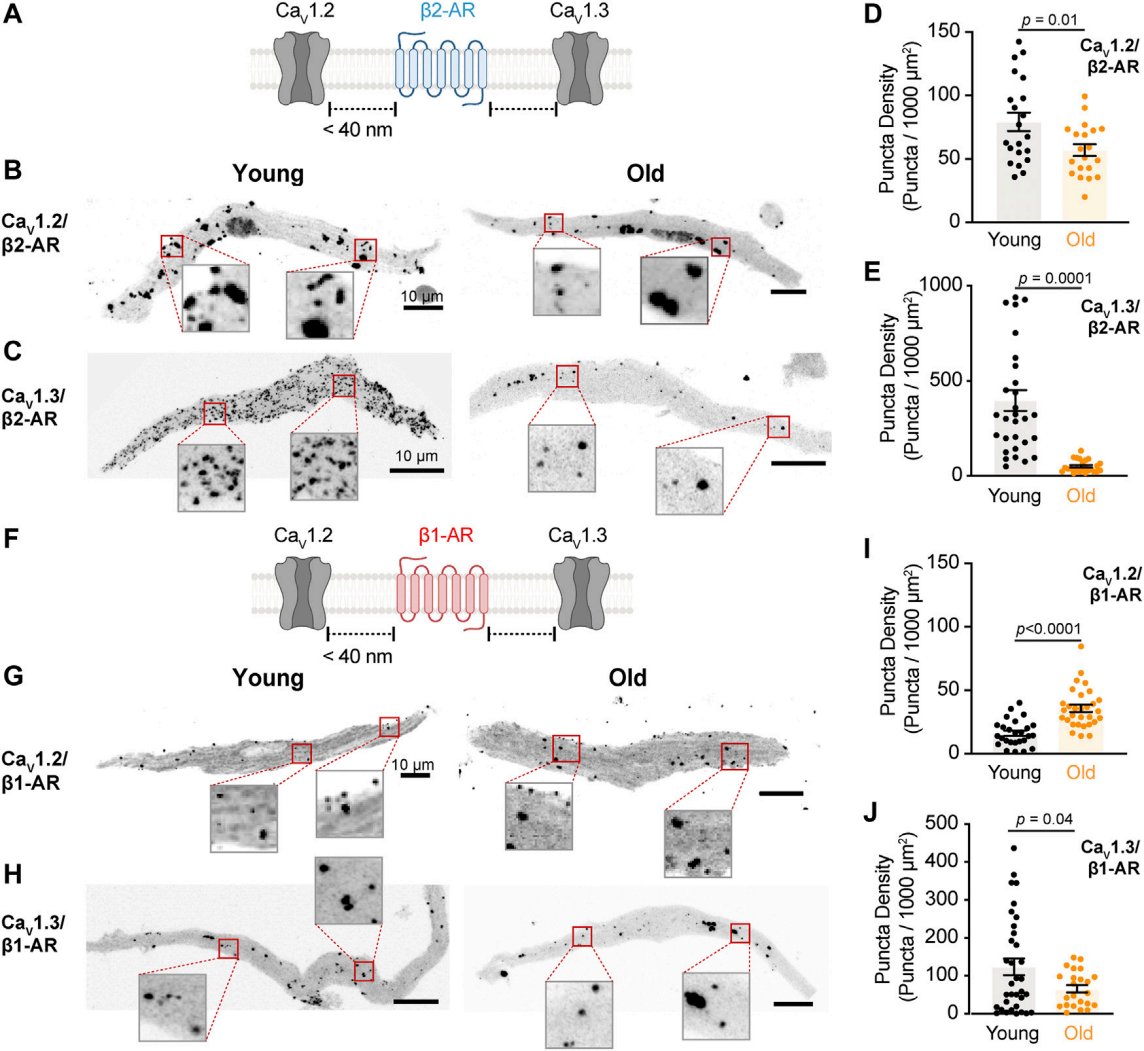
The physical association between L-type calcium channels and β2-AR is reduced in old pacemaker cells. **(A)** Diagrammatic representation of the molecular association being tested between β2-AR and specific LTCCs. Diagram created with BioRender.com. **(B)** Representative pictures of proximity ligation assay (PLA) between β2-AR and Ca_V_1.2 channels in young and old pacemaker cells. **(C)** Representative PLA pictures between β2-AR and Ca_V_1.3 channels in young and old pacemaker cells. **(D)** Comparison of the puncta density formed by the association of β2-AR with Ca_V_1.2 channels between young (n = 21 cells, N = 3 mice) and old (n = 20 cells, N = 3 mice). **(E)** Comparison of the puncta density formed by the association of β2-AR with Ca_V_1.3 channels between young (n = 30 cells, N = 3 mice) and old (n = 24 cells, N = 3 mice). **(F)** Diagrammatic representation of the molecular association being tested between β1-AR and specific LTCCs. **(G)** Representative PLA pictures between β1-AR and Ca_V_1.2 channels in young and old pacemaker cells. **(H)** Representative PLA pictures between β1-AR and Ca_V_1.3 channels in young and old pacemaker cells. **(I)** Comparison of the puncta density formed by the association of β1-AR with Ca_V_1.2 channels between young (n = 26 cells, N = 3 mice) and old (n = 30 cells, N = 3 mice). **(J)** Comparison of the puncta density formed by the association of β1-AR with Ca_V_1.3 channels between young (n = 34 cells, N = 3 mice) and old (n = 23 cells, N = 3 mice). Statistical comparison used an unpaired t-test with significance at *p* < 0.05.

As the expression of β-ARs did not change, we tested whether the molecular proximity between the two specific β-AR isoforms and L-type calcium channels were disrupted in old cells using Proximity Ligation Assay (PLA, Figure 3A). The formation of PLA fluorescent puncta between a specific pair of proteins (β2-AR/Ca_V_1.2, β2-AR/Ca_V_1.3, β1-AR/Ca_V_1.2, and β1-AR/Ca_V_1.3) was evaluated in isolated pacemaker cells from young and old mice. The formation of fluorescent puncta was interpreted as proximity below 40 nm between the two proteins of interest and it was quantified as the puncta number normalized by cell area (puncta/1,000 μm^2^). PLA quantifications were expressed as puncta density regardless of puncta size since different factors can influence the size of the fluorescent puncta including variation in the ligation and amplification efficiency, antibody type, and the number of bound antibodies per protein. Negative controls, where one of the two primary antibodies was omitted, resulted in a low puncta density (Supplementary Figure S1), confirming that the pairs of secondary probes used do not bind close to each other non-specifically. We first tested the changes in the association between the β2-AR and Ca_V_1.2 channels. Puncta density was significantly lower in old (56.9 ± 4.6, n = 20) compared to young cells (79.2 ± 7.2, n = 21) (Figures 3B,D). A more dramatic reduction was observed when comparing puncta density between β2-AR and Ca_V_1.3 channels in young and old cells. In old pacemaker cells, the β2-AR/Ca_V_1.3 puncta density was seven-fold smaller than in young cells (50.7 ± 6.6, n = 24 vs. 369.9 ± 54.5, and n = 30, Figures 3C,E). Next, we used PLA to assess the effect of aging on the molecular proximity between β1-ARs and L-type calcium channels (Figure 3F). In contrast to the reduction observed for the β2-ARs, pacemaker cells co-labeled with primary antibodies against β1-AR and Ca_V_1.2 channels showed a significantly higher puncta density in old animals compared to that of young (35.8 ± 2.8, n = 30 vs. 16.1 ± 2.0, and n = 26, Figures 3G,I). A small reduction was observed when comparing the puncta density between β1-AR and Ca_V_1.3 channels. For β1-AR/Ca_V_1.3 the puncta density was 65.7 ± 9.8 (n = 23) in old cells, while in young cells, the puncta density was 123.7 ± 21.7 (n = 34, Figures 3H,J). The specific reduction in the proximity of β2-AR and L-type calcium channels observed in old cells correlates with the specific decrease in the calcium current upregulation mediated by the activation of β2-ARs with formoterol. Altogether, these results suggest that the β2-mediated regulation of L-type calcium channels is compromised in pacemaker cells of old animals due to loss of proximity between β2-AR and both Ca_V_1.2 and Ca_V_1.3 channels rather than a reduction in cellular β2-AR abundance.

### Aging Reduces the Association of Ca_V_1.2 and Ca_V_1.3 Channels With the Scaffolding Protein AKAP150 in Pacemaker Cells

**FIGURE 4:**
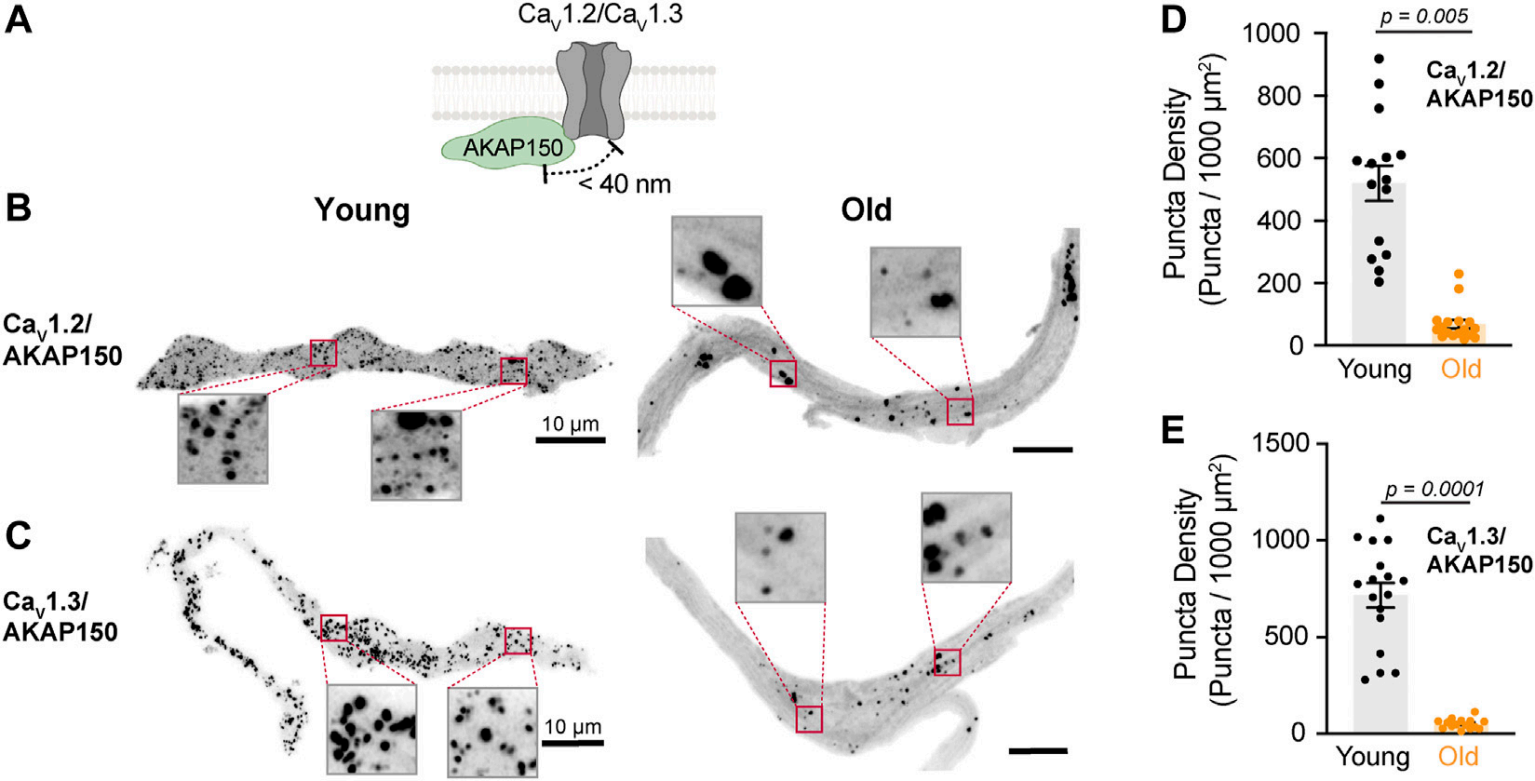
Aging reduces the association between L-type calcium channels and AKAP150. **(A)** Diagrammatic representation of the molecular association being tested between AKAP150 and specific LTCCs. Diagram created with BioRender.com. **(B)** Representative PLA pictures between AKAP150 and Ca_V_1.2 channels in young and old pacemaker cells. **(C)** Representative PLA pictures between AKAP150 and Ca_V_1.3 channels in young and old pacemaker cells. **(D)** Comparison of the puncta density formed by the association of AKAP150 with Ca_V_1.2 between young (n = 15, N = 3 mice) and old (n = 17, N = 3 mice) pacemaker cells. **(E)** Comparison of the puncta density formed by the association of AKAP150 with Ca_V_1.3 channels between young (n = 15, N = 3 mice) and old (n = 17, N = 3 mice) pacemaker cells. Statistical comparison used an unpaired t-test with significance at *p* < 0.05.

Scaffolding proteins play an important role in recruiting and maintaining the molecular components inside signaling microdomains. Little is known about what scaffolding proteins maintain β-adrenergic signaling microdomains in pacemaker cells. AKAP150 is perhaps the most studied scaffolding protein in the cardiovascular system. In ventricular cardiomyocytes (Cheng et al., 2011) and vascular smooth muscle cells (Navedo et al., 2008), AKAP150 has been shown to bind β-ARs and L-type calcium channels. The function of AKAP150 in pacemaker cells has yet to be studied. We used PLA in isolated pacemaker cells to determine if L-type calcium channels are associated with AKAP150 and determine whether aging changes this association (Figure 4A). We found that aging causes a dramatic reduction in the proximity between AKAP150 and both channels. AKAP150-Ca_V_1.2 puncta density was reduced by 7-fold in old cells, from 520.0 ± 56.3 puncta in young (n = 15) to 68.4 ± 13.6 puncta in old (n = 17) (Figures 4B,D). The association between AKAP150 and the Ca_V_1.3 channel was reduced even further, showing a 14-fold reduction in puncta density, going from 716.0 ± 63.1 puncta in young (n = 15) to 51.4 ± 6.7 puncta in old cells (n = 17) (Figures 4C,E). The observed strong association of AKAP150 with both L-type calcium channels suggests that AKAP150 plays an important role in anchoring these channels in pacemaker cells and that this association is susceptible to the deleterious effects of aging.

### Aging Decreases Caveolar Density in Pacemaker Cells and the Recruitment of L-type Calcium Channels Into These Microdomains

Caveolae are abundant in pacemaker cells and enable the local signaling of β-ARs due to the compartmentalization of different effectors such as L-type calcium channels (Barbuti et al., 2007).

**FIGURE 5:**
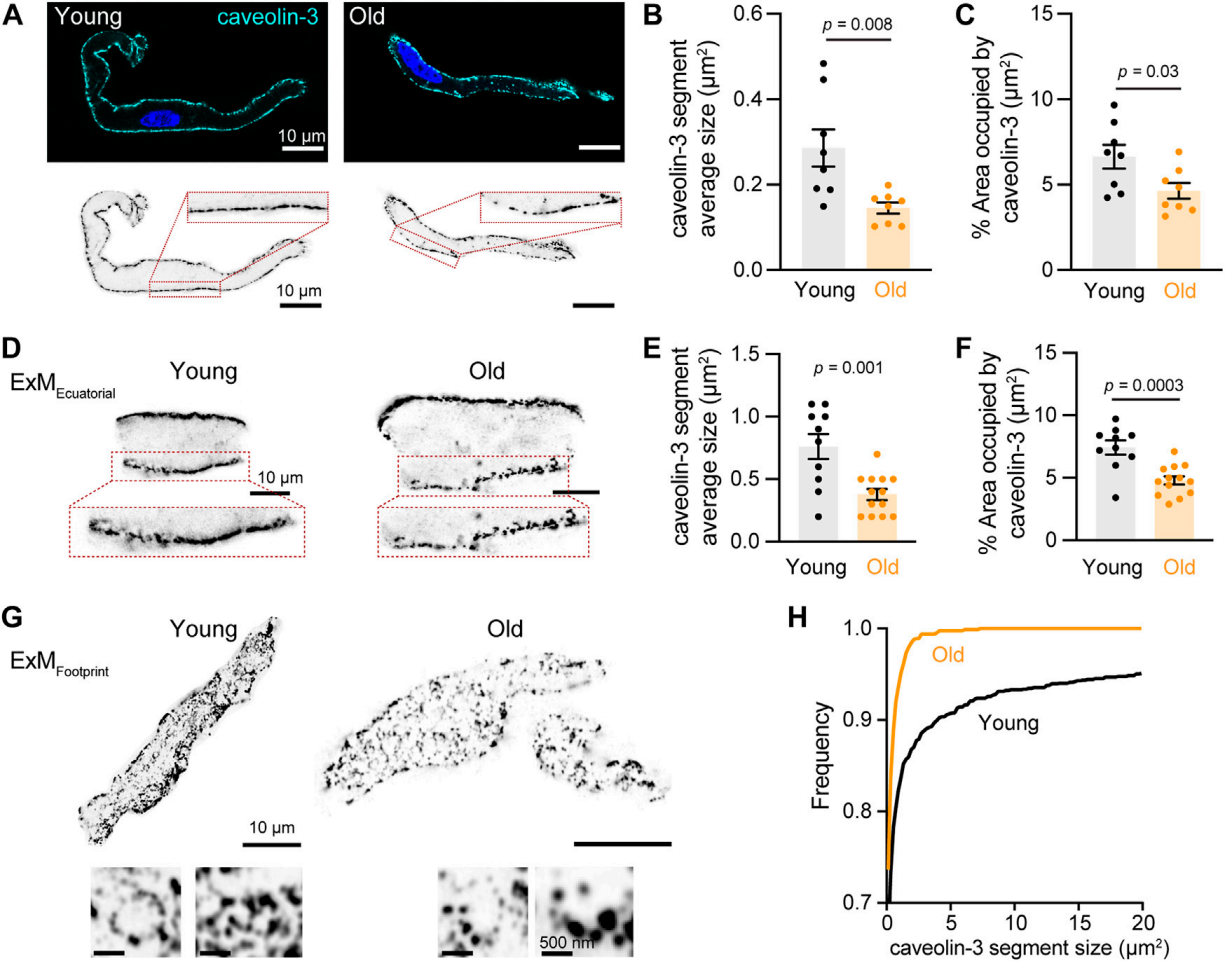
Aging decreases the size and area occupied by caveolae. **(A)** Representative pictures of pacemaker cells labeled with antibody anti-caveolin-3 (cyan) and counterstained with DAPI to visualize the nucleus (blue). Same pictures in grayscale and inverted are shown at the bottom. **(B)** Comparison of the average segment size positive for caveolin-3 between young (n = 8, N = 3 mice) and old (n = 8, N = 3 mice) cells. **(C)** Comparison of percent area occupied by caveolin-3 structures between young (n = 8, N = 3 mice) and old (n = 8, N = 3 mice) cells. **(D)** Representative pictures of pacemaker cells expanded to improve the resolution of caveolin-3 segments. Only a region of the cell is shown. **(E)** Comparison of the average segment size positive for caveolin-3 between expanded young (n = 10, N = 3 mice) and old (n = 13, N = 3 mice) cells. **(F)** Comparison of percent area occupied by caveolin-3 structures between expanded young (n = 10, N = 3 mice) and old (n = 13, N = 3 mice) cells. Note a 2.5-fold increase in segment size between expanded and not expanded cells. Statistical comparison used an unpaired t-test with significance at *p* < 0.05. **(G)** Representative pictures of the footprint of expanded young and old pacemaker cells labeled with anti-caveolin-3. **(H)** Comparison of the cumulative frequency distribution of the size of caveolin-3 segments from expanded young (black, n = 10, N = 3 mice) and old (orange, n = 13, N = 3 mice) cells.

We assessed whether old cells exhibit less or disrupted caveolae by immunostaining against caveolin-3 and imaging with high-resolution microscopy. Young cells showed continuous caveolin-3 labeling along the plasma membrane, consistent with the high density of caveolae in these cells. However, in old cells, the membrane labeling of caveolin-3 had a discontinuous dash-like pattern (Figure 5A). To quantify this alteration, we set thresholds and binarized the signal to isolate the labeling at the plasma membrane of the equatorial planes of the cell. The size of caveolin-3 positive segments was significantly shorter in old (0.14 ± 0.01 μm^2^, n = 8) compared to young cells (0.29 ± 0.04 μm^2^, n = 8) (Figure 5B). The percentage area occupied by caveolin-3 was smaller in old (4.63 ± 0.46%, n = 8) compared to young cells (6.64 ± 0.70%, n = 8) (Figure 5C). As the external diameter of caveolae is only about 70 nm (Matthaeus and Taraska, 2020), we utilized expansion microscopy combined with high-resolution imaging to determine the differences in caveolae size between young and old cells. With this improved resolution, the reduction in caveolae density at the plasma membrane in old cells was even more evident (Figure 5D). The size of caveolin-3 positive structures in old cells was half of that observed in young cells (0.38 ± 0.04 μm^2^, n = 13 vs. 0.76 ± 0.10 μm^2^, and n = 10, Figure 5E). Moreover, the area occupied by caveolin-3 was also smaller in expanded old cells (4.8 ± 0.3%, n = 13) compared to expanded young cells (7.4 ± 0.6%, n = 10) (Figure 5F). The labeling of caveolin-3 at the cell footprint exhibited a bimodal pattern between punctuated and reticular (Figure 5G). By analyzing these images, we confirmed that the caveolar size distribution was shifted toward smaller sizes in old compared to young cells (Figure 5H).

**FIGURE 6:**
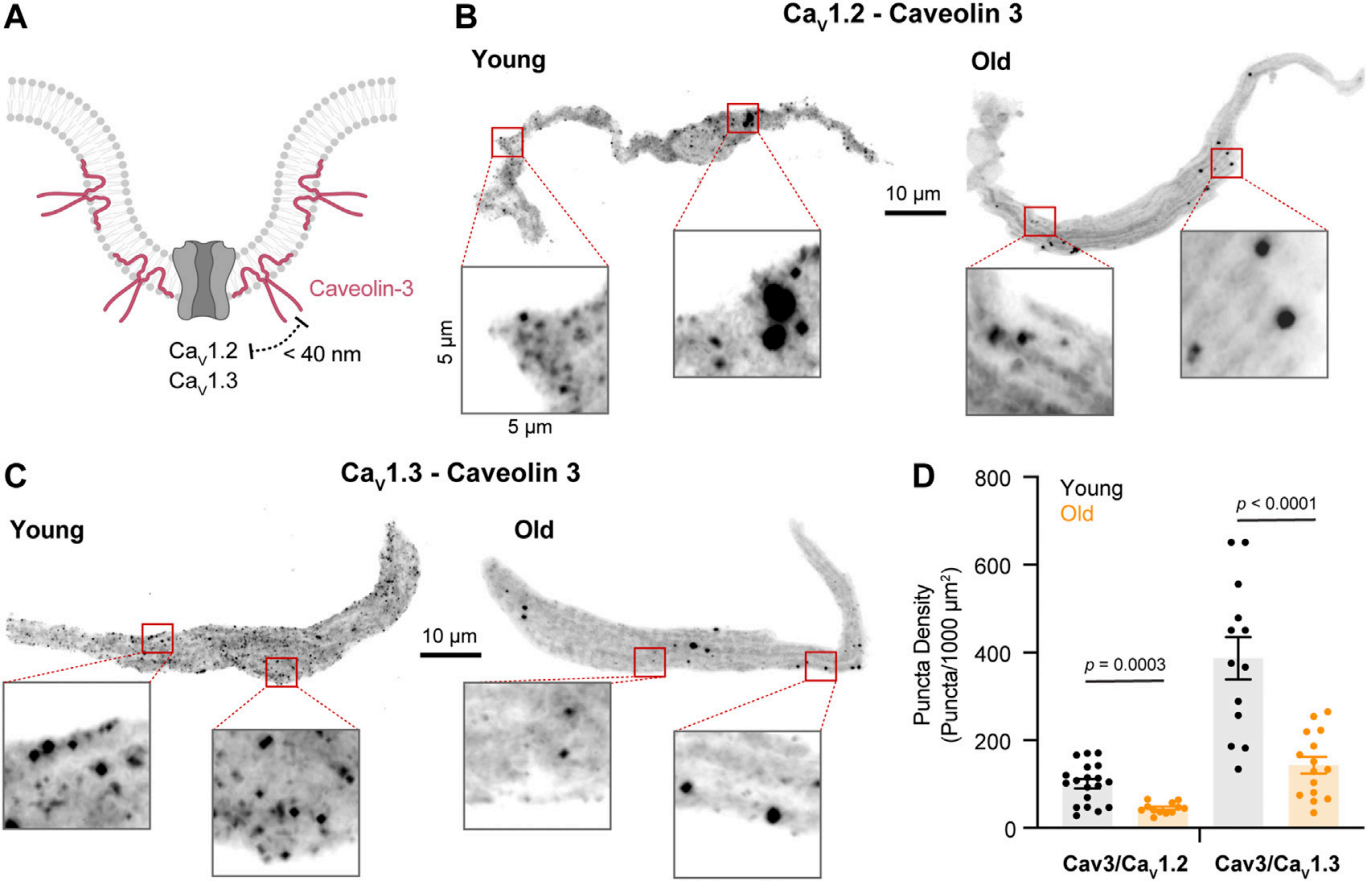
Aging decreases the association between L-type calcium channels and caveolin-3. **(A)** Diagrammatic representation of the molecular association being tested between caveolin-3 and specific LTCCs. Diagram created with BioRender.com. **(B)** Representative PLA pictures between caveolin-3 and Ca_V_1.2 channels in young and old pacemaker cells. **(C)** Representative PLA pictures between caveolin-3 and Ca_V_1.3 channels in young and old pacemaker cells. **(D)** Comparison of the puncta density formed by the association of caveolin-3 with specific LTCCs in young (Ca_V_1.2 n = 19 cells, Ca_V_1.3 n = 13 cells, and N = 3 mice) and old (Ca_V_1.2 n = 12 cells, Ca_V_1.3 n = 15 cells, and N = 3 mice). Statistical comparison used an unpaired t-test with significance at *p* < 0.05.

Although caveolae size (as reported by caveolin-3) was reduced in old cells, L-type calcium channels could still be strongly associated with these fragmented structures. Therefore, we decided to evaluate the association between caveolin-3 and L-type calcium channels (Figure 6A). We first used PLA in isolated pacemaker cells co-labeled with primary antibodies against caveolin-3 and Ca_V_1.2 channels. The PLA fluorescent puncta density was 2-times lower in old (45.08 ± 3.63, n = 12) compared to young (101.1 ± 10.4, n = 19) (Figures 6B,D). In pacemaker cells co-labeled with primary antibodies against caveolin-3 and Ca_V_1.3 channels, the PLA fluorescent puncta density was almost 3-times lower in old (143.5 ± 19.1, n = 15) compared to young (387 ± 47.9, n = 13) (Figures 6C,D). Altogether, these results suggest that in pacemaker cells, aging is associated with a reduction in caveolae and a loss of caveolar localization of L-type calcium channels.

## Discussion

Adrenergic modulation has two main effects on the heart: the first one is to increase ventricular contraction force, also known as the positive inotropic effect, and the second one is to accelerate pacemaker rate, known as the positive chronotropic effect. Studies in ventricular cardiomyocytes have revealed that the activation of β1 and β2-ARs play different roles in the positive inotropic effect induced by noradrenaline (Xiao and Lakatta, 1993; Altschuld et al., 1995; Kuschel et al., 1999). These differences rely on the compartmentalization of β-AR receptors into different signaling microdomains and their specific association with sub-pools of Ca_V_1.2 channels (Johnson and Antoons, 2018). While this has been extensively studied in the context of ventricular inotropy (Nikolaev et al., 2010; Wright et al., 2014; Schobesberger et al., 2017), the characteristics of this subtype-specific association in pacemaker cells and its role in the positive chronotropic effect is much less understood. The fact that pacemaker cells express a higher ratio of β2-AR compared to other cardiac regions has suggested that β2-ARs may have an important role in the chronotropic effect. Moreover, the fact that pacemaker cells express both Ca_V_1.2 and Ca_V_1.3 channels also opens the question of whether these channels associate preferentially to any of the β-AR subtypes or if they are differentially regulated by them. Our results show that in fact, in young pacemaker cells both L-type calcium channels are preferentially associated to β2-ARs. In contrast with previous results (Larson et al., 2013), when we simultaneously activated both β-ARs using the non-specific agonist isoproterenol, we observed that upregulation of the calcium current in old cells is significantly reduced compared to that of young cells. However, when we dissected the β1 and β2 components pharmacologically, we unveiled that aging specifically affects the L-type calcium current upregulation mediated by the β2-AR. No significant changes were observed for the upregulation mediated by the β1-AR. However, it would be interesting to measure dose-response curves for each receptor to determine if aging has any effect on the receptor sensitivity that is not evident at the saturating concentrations used in this study.

This reduction is in part explained by an age-associated reduction in the physical proximity between Ca_V_1.2 and Ca_V_1.3 channels and β2-ARs. Our results agree with the idea put forward by DiFrancesco’s group that the compartmentalization and the specific stimulation of β2-ARs are the main mechanisms by which heart rate is modulated (DiFrancesco, 1986). Interestingly, the association with β2-ARs and the age-associated reduction of this association was remarkably more robust for the Ca_V_1.3 than for the Ca_V_1.2 channel. It had been previously proposed that in pacemaker cells, the Ca_V_1.2 channel was the one preferentially associated with β2-ARs (Balijepalli et al., 2006; Harvey and Hell, 2013), similar to what has been seen in ventricular cardiomyocytes (Lang and Glukhov, 2018). However, we show that this is not the case. This finding is relevant since Ca_V_1.3 channels not only contribute to the action potential of the pacemaker but they also play a crucial role in the diastolic depolarization that drives pacemaker automaticity (Christel et al., 2012). Therefore, their close association with β2-ARs suggests that Ca_V_1.3 channels are one of the main targets for the adrenergic control of pacemaker rate. It also shows that the reduction of Ca_V_1.3 regulation is an important mechanism in the age-associated loss of responsivity to adrenergic stimulation.

Are other components of the β-AR and L-type calcium channel signaling microdomains disrupted by aging? In the case of β-adrenergic signaling in ventricular cardiomyocytes, scaffolding proteins play an important role in recruiting and organizing specific molecular components into these microdomains. AKAP150 (Cong et al., 2001; Liu et al., 2004; Tao and Malbon, 2008) and AKAP250 (Shih et al., 1999; Tao et al., 2003; Malbon et al., 2004) were initially identified as partners for the β2-ARs. The binding of β2-ARs and Ca_V_1.2 channels to AKAP150 is essential for the localization of these molecules to T-tubules and the correct excitation-contraction coupling (Balijepalli et al., 2006; Nichols et al., 2010). In contrast, the association of β1-ARs and a different pool of Ca_V_1.2 channels to AKAP150 outside T-tubules plays an important role in the generation of global cAMP elevations. Our results indicate that AKAP150 strongly associates with Ca_V_1.2 and Ca_V_1.3 channels in pacemaker cells and that aging causes a dramatic reduction in this association. However, whether these associations are happening preferentially inside or outside caveolar microdomains need to be further studied. It is unknown what specific scaffolding proteins organize β-ARs and L-type calcium channels in pacemaker cells. Mutations in different scaffolding proteins including AKAP10 (Tingley et al., 2007), ankyrin-B (Mohler et al., 2003; Le Scouarnec et al., 2008), and caveolin-3 (Campostrini et al., 2017) have been seen to cause bradycardia and other forms of pacemaker dysfunctions (Lang and Glukhov, 2018). Thus, future research should focus on systematically testing for the role of these scaffolding proteins in the formation of β-ARs/L-type calcium channels microdomains and their alterations in aging.

In ventricular cardiomyocytes, β2-AR are compartmentalized in close proximity to Ca_V_1.2 channels inside caveolae and t-tubules. As a result, the cAMP elevations triggered by the activation of β2-AR are localized, and their main function is to precisely facilitate the calcium current without affecting other PKA-dependent processes (Kuschel et al., 1999; Xiao et al., 1999). Pacemaker cells lack T-tubules, and caveolae have been proposed to serve as compartments for the signaling between β2-ARs and L-type calcium channels. β2-AR receptors in pacemaker cells have been seen to localize preferentially inside caveolae in close proximity to HCN4 channels (Barbuti et al., 2012). Our results show that caveolae also facilitate the close proximity of β2-AR receptors to Ca_V_1.3 and Ca_V_1.2 channels. Interestingly, our results not only demonstrate that L-type calcium channels associate with caveolin-3 but that this association is reduced with age. We also show that aging causes a decrease in caveolae density in pacemaker cells. These findings are in line with reports in ventricular cardiomyocytes from patients with heart disease (Fridolfsson and Patel, 2013; Egorov et al., 2019). A limitation of our study is that it was not possible to test the changes in proximity between β-ARs and caveolin-3. More data is necessary to estimate the ratio of β2-ARs that locate inside and outside caveolae and the effect of aging on this ratio. The important role of caveolae in cardiac function has been highlighted by the fact that animal models lacking caveolin-1 and -3 display severe cardiomyopathy characterized by an increase in ventricular wall thickness, hypertrophy, and a decrease in fractional shortening. In addition, mutations in caveolin-3 are associated with long-QT congenital syndrome (Vatta et al., 2006). The importance of caveolin-3 in the specific function of the pacemaker has been shown in the caveolin-3 cardiac-specific KO mouse, where there is a significant beat-to-beat heart rate lability linked with suppressed pacemaker function (Lang and Glukhov, 2018). Here, we reveal that the age-associated disruption of caveolae is a newly identified mechanism that leads to a reduction in the compartmentalized β-adrenergic signaling and in the responsiveness of the pacemaker to this modulation.

**FIGURE 7:**
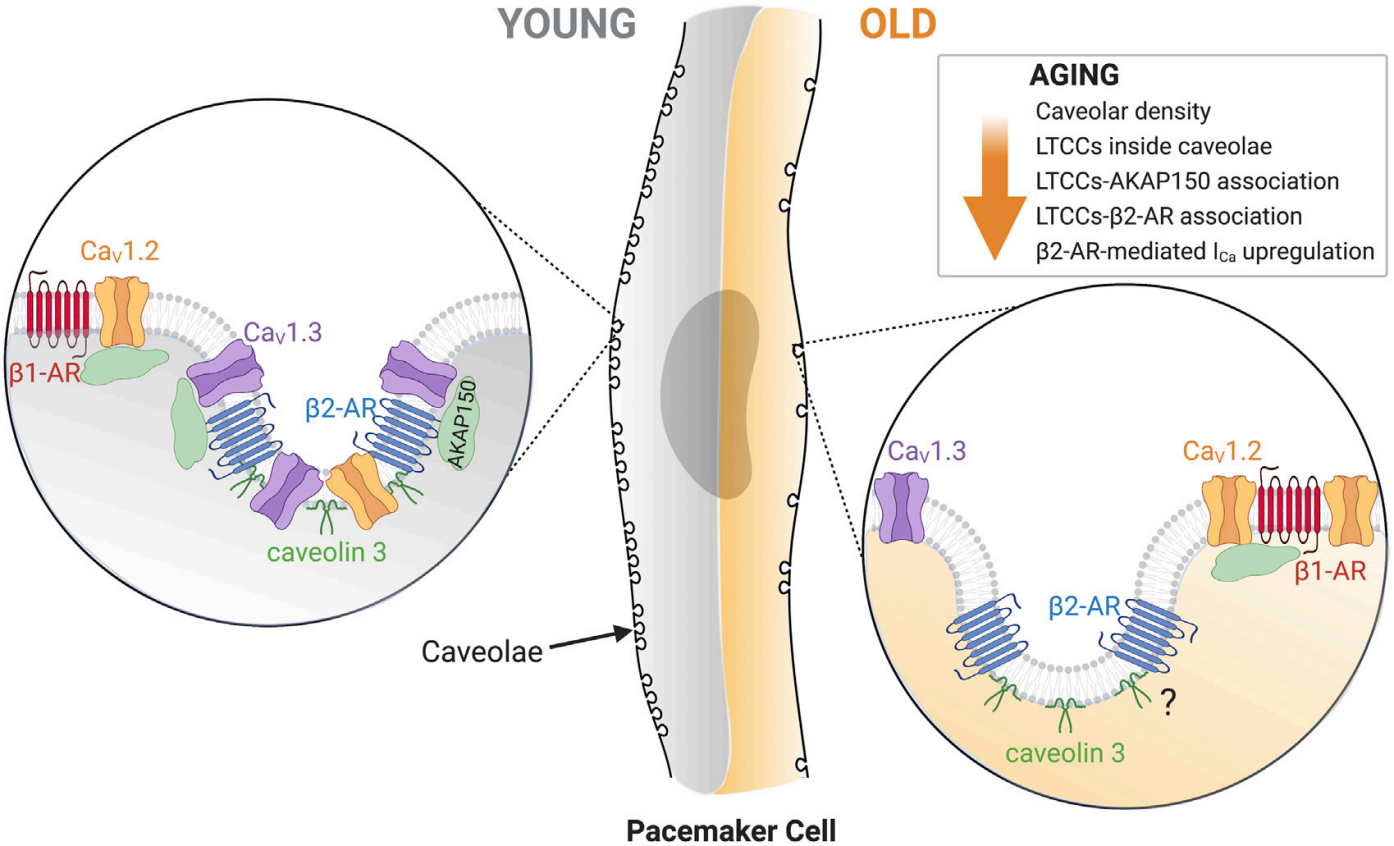
Diagrammatic representation of the main changes in old pacemaker cells that lead to a reduction in L-type calcium current regulation. A global reduction in caveolar density is characteristic of old cells. Zoomed in on the left side represent the proposed architectural layout of associated proteins in young cells: β1-AR (red)/Ca_V_1.2 (orange) outside of caveolae and β2-AR (blue)/Ca_V_1.2 (orange)/Ca_V_1.3 (purple)/Caveolin-3 (dark green)/AKAP150 (light green) inside caveolae. Zoomed in on the right side shows the proposed architectural changes of associated proteins in old cells: β1-AR (red)/Ca_V_1.2 (orange) remain outside caveolae, and β2-AR (blue)/Caveolin-3 (dark green) remain inside caveolae, while Ca_V_1.3 shift outside caveolae. If aging alters the proximity of β2-AR with caveolin-3 needs future testing. Created with BioRender.com.

Figure 7 summarizes our model for the age-associated impairment of β2-mediated L-type calcium current regulation. We propose that young pacemaker cells have a vast caveolar network that allows efficient signaling between β2-ARs and L-type calcium channels. In these networks, L-type calcium channels are anchored through their binding to AKAP150. Ca_V_1.3 channels are especially enriched inside these caveolar microdomains, while Ca_V_1.2 channels can also associate with β1-ARs outside these microdomains. In contrast, old pacemaker cells exhibit a global reduction in caveolar density. This is accompanied by a reduction in the co-localization of Ca_V_1.3 and Ca_V_1.2 channels with β2-ARs inside caveolae. However, whether β2-ARs compartmentalization inside caveolae is reduced with age has yet to be tested. As HCN4 channels, NCX, and potassium channels are also localized inside caveolae and modulated by β2-ARs (Lang and Glukhov, 2018), future studies will have to evaluate the effects of the age-associated reduction in caveolar density over the function of these proteins to gain a more holistic view on how aging affects pacemaker dysfunction at the molecular level.

## Data Availability

The raw data supporting the conclusion of this article will be made available by the authors, upon request.
