# Supplementary material for: Aging Alters the Formation and Functionality of Signaling Microdomains Between L-type Calcium Channels and β2-Adrenergic Receptors in Cardiac Pacemaker Cells
Source: Front Physiol. 2022 Apr 20;13:805909. doi: 10.3389/fphys.2022.805909 (PMC9065441; doi:10.3389/fphys.2022.805909)
Supplement: Supplementary file 1 [file Presentation1.pdf]

SUPPLEMENTARY MATERIAL

	Control condition		
	Young (n=6)	Old (n=7)	Unpaired t-test
AP frequency (bpm)	187.2±9.2	111.9 ± 13.9	**
Cycle length (ms)	324.4 ±15.4	623.8 ±97	*
SD of CL (ms)	46.2 ± 17.64	183.4 ± 73.3	ns
MDP (mV)	-59.89±2.3	-56.03±0.8	ns
APA (mV)	98.08±7.8	91.4±8.9	ns
Eth (mV)	-36.45±1.9	-30.91±0.9	*
APD (ms)	183.4±23.9	259.6±23.5	*
APD70 (ms)	74.6±11.4	92.35±6.5	ns
APD90 (ms)	108.4±13.7	147.1±9.2	*
RD (ms)	175.1±19.2	254.8±22.9	*
DD duration (ms)	127 ± 8.9	313.7±73.2	*
DD amplitude (mV)	23±1	24.5±0.8	ns
Slope EDD (mV/ms)	0.10±0.007	0.02±0.007	****
Slope LDD (mV/ms)	1.4±0.10	0.43±0.07	****
DV/dt (mV/ms)	35.8±8.5	35±11.2	ns

Supplementary Table 1. Parameters of spontaneous APs from young and old pacemaker cells. Comparison of the AP parameters in young and old pacemaker cells. Spontaneous APs were recorded in β-escin perforated-patch clamp. Data represents an average of 3 measurements per trace and is presented as mean ± SEM. Statistical comparisons used an unpaired t-test with significance at $p<0.05$. Abbreviations and description of measurements: Cycle length was measured between two AP; SD of CL (standard deviation of the cycle length) describes AP variability; MDP (maximal diastolic potential) was the lowest membrane potential measured within the DD; DD (diastolic depolarization); APA (action potential amplitude) was measured from the MDP to the AP peak; Eth (AP upstroke threshold) was determined as the end point of the DD phase; APD (AP duration) was measured as the time from the Eth to the next MDP, APD70 was measured as the time from the Eth to the point measured at 70% of the repolarization; APD90 was measured as the time from the Eth to the point measured at 90% of the repolarization; RD (repolarization duration) was measured as the time from AP peak to the next MDP, the DD duration was calculated from MDP to Eth, DD amplitude was calculated from MDP to Eth, the slope of the early phase of DD (EDD) was calculated as the linear slope of DD; the slope of the late phase of DD (LDD) was calculated as the exponential phase of the DD; dV/dt was calculated as the slope of the AP upstroke measured from the Eth to the AP peak.

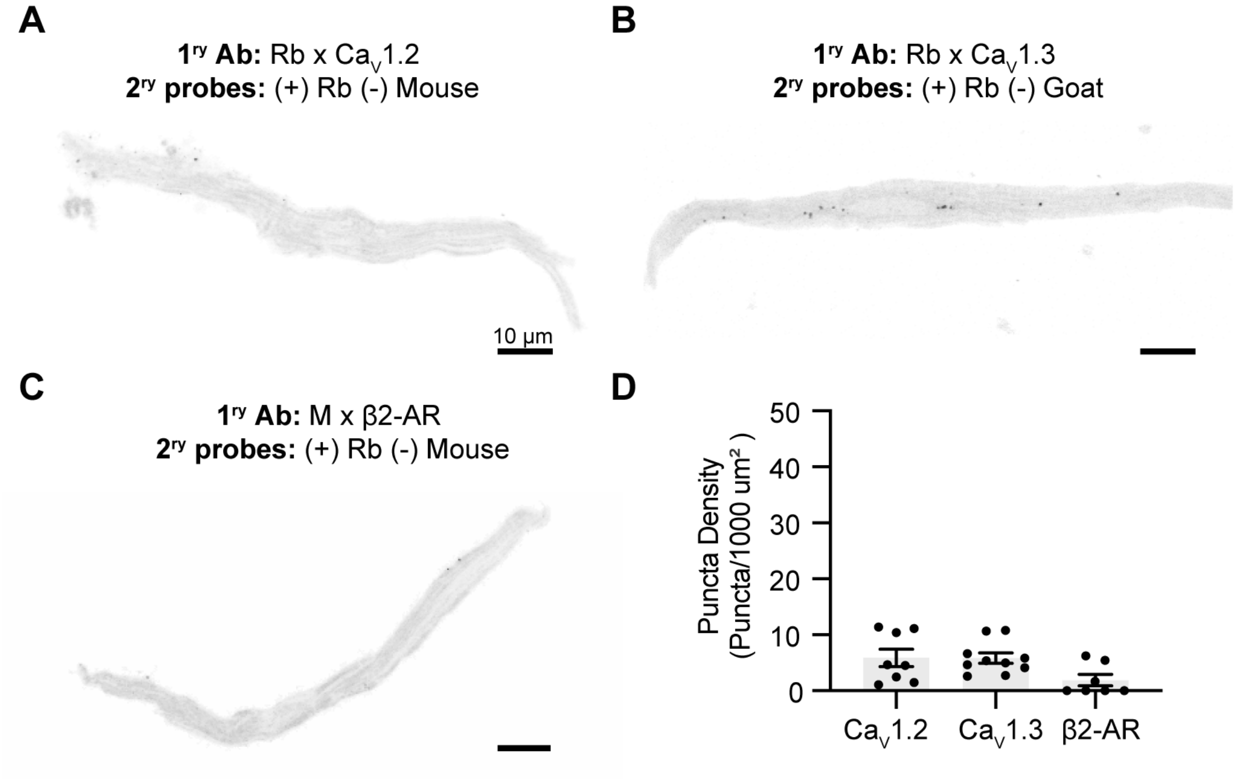

Supplementary Figure 1. Negative controls for proximity ligation assays. (A) Representative picture of a negative control for the proximity ligation assay (PLA) using a Rabbit (Rb) antibody against $Ca_V1.2$ channels with no antibody against any interacting partner. Secondary antibodies against Rb and mouse primary antibodies were added to the assay. (B) Representative negative PLA picture using a Rb antibody against $Ca_V1.3$ channels with no antibody against any interacting partner. Secondary antibodies against Rb and goat primary antibodies were added to the assay. (C) Representative negative PLA picture using a mouse antibody against β2-AR with no antibody against any interacting partner. Secondary antibodies against Rb and mouse primary antibodies were added to the assay. (D) Comparison of the puncta density observed in the negative controls as shown in panels A to C. n = 8 cells for $Ca_V1.2$, n = 10 cells for $Ca_V1.3$, and n = 7 cells for β2-AR. N = 2 mice in all experiments. Statistical comparison used an unpaired t-test with significance at $p<0.05$.
