# Supplementary figures and images for: Aging Alters the Formation and Functionality of Signaling Microdomains Between L-type Calcium Channels and β2-Adrenergic Receptors in Cardiac Pacemaker Cells
Source: Front Physiol. 2022 Apr 20;13:805909. doi: 10.3389/fphys.2022.805909 (PMC9065441; doi:10.3389/fphys.2022.805909)

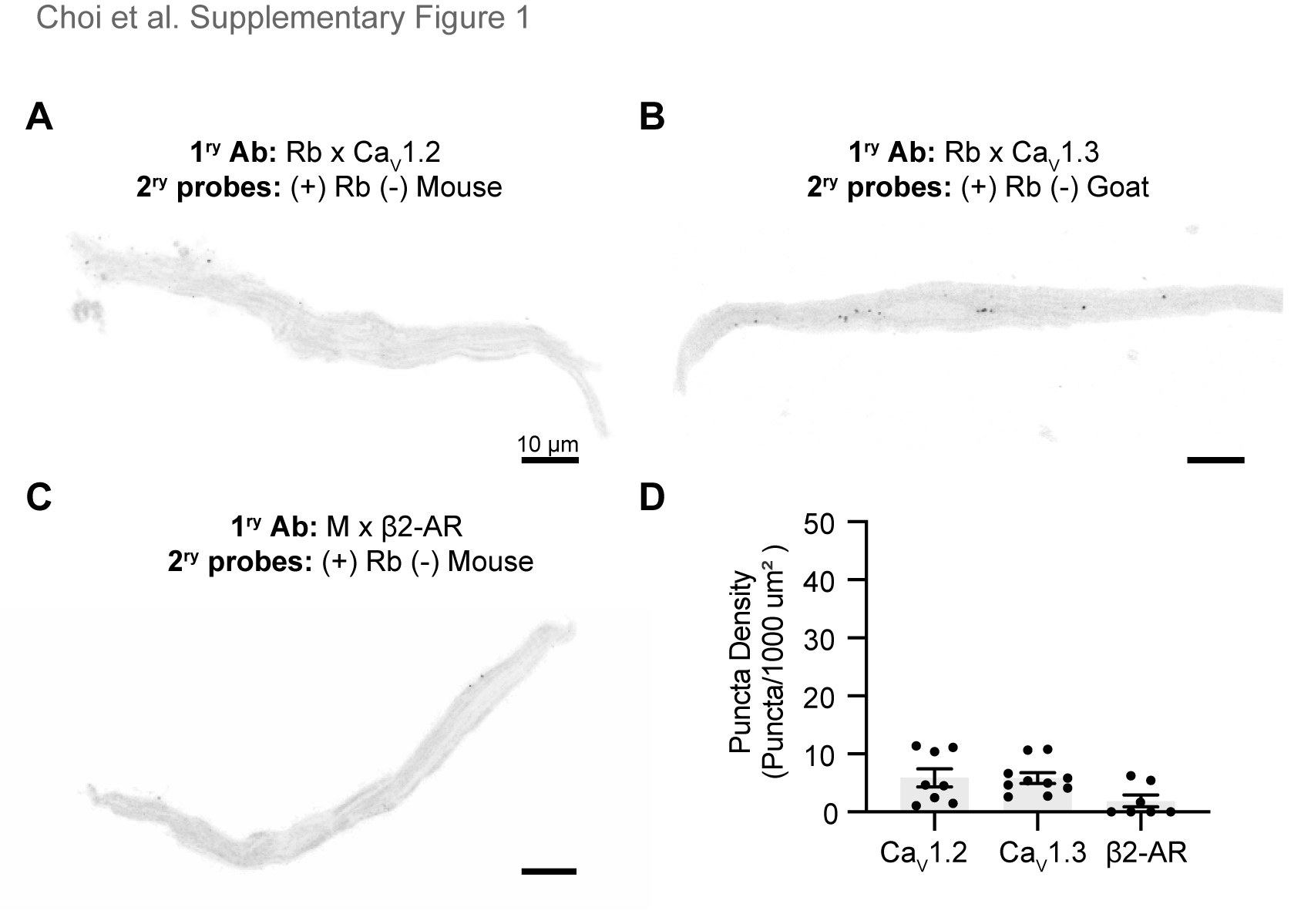

Supplement: Supplementary file 2 [file Image1.TIF]
